# Improving Maternal and Reproductive Health in Kigoma, Tanzania: A 13-Year Initiative

**DOI:** 10.9745/GHSP-D-21-00484

**Published:** 2022-04-28

**Authors:** Neena Prasad, Nguke Mwakatundu, Sunday Dominico, Prudence Masako, Wilfred Mongo, Yisambi Mwanshemele, Godson Maro, Leonard Subi, Paul Chaote, Neema Rusibamayila, Alicia Ruiz, Karen Schmidt, Mkambu Godfrey Kasanga, Samantha Lobis, Florina Serbanescu

**Affiliations:** a Bloomberg Philanthropies, New York, NY, USA.; b Thamini Uhai, Dar es Salaam, Tanzania.; c EngenderHealth, Dar es Salaam, Tanzania.; d Ministry of Health, Community Development, Gender, Elderly and Children, Dodoma, Tanzania.; e President's Office Regional Administration and Local Government, Health Social Welfare and Nutrition Division, Dodoma, Tanzania.; f U.S. Centers for Disease Control and Prevention, Division of Reproductive Health, Atlanta, USA.; g Vital Strategies, New York, USA.

## Abstract

The Program to Reduce Maternal Deaths in Tanzania was a 13-year (2006–2019) effort in the Kigoma region that evolved over 3 phases to improve and sustain the availability of, access to, and demand for high-quality maternal and reproductive health care services. The Program intended to bring high-quality care closer to more communities. Cutting across the Program was the routine collection of monitoring and evaluation data. The Program achieved significant reductions in maternal and perinatal mortality, a significant increase in the modern contraceptive prevalence rate, and a significant decline in the unmet need for contraception. By 2017, it was apparent that the Program was on track to meet or surpass many of the targets established by the Government of Tanzania. Over the following 2-plus years, efforts to sustain Program interventions intensified. In April 2019, the Program fully transitioned to Government of Tanzania oversight. Four key lessons were learned during implementation that are relevant to governments, donors, and implementing organizations working to reduce maternal mortality: (1) multistakeholder partnerships are critical; (2) demand creation for services, while critical, must rest on a foundation of well-functioning and high-quality clinical services; (3) it is imperative to not only collect robust monitoring and evaluation data, but to be responsive in real time to what the data reveal; and, (4) it is necessary to develop a deliberate sustainability strategy from the start. The Program in Kigoma demonstrates that decentralizing high-quality maternal and reproductive health services in remote, low-resource settings is both feasible and effective and should be considered in places with similar contexts. By embedding the Program in the existing health system, and through efforts to build local capacity, the improvements seen in Kigoma are likely to be sustained. Follow-up evaluations are planned, providing an opportunity to more directly assess sustainability.

## Abstract (precis)

The 13-year Program to Reduce Maternal Deaths in Tanzania employed multifaceted maternal, newborn, and reproductive health interventions that contributed to increasing the availability and utilization of high-quality obstetric and family planning services and reducing maternal and perinatal mortality in Kigoma.

## Teaser

**Key Findings:**

- The Program in Kigoma demonstrated that decentralizing high-quality maternal and reproductive health services to lower-level facilities in remote, low-resource settings is both feasible and effective.
- Between 2013 and 2018, Kigoma achieved significant reductions in maternal and perinatal mortality and increases in contraceptive prevalence by improving the availability of and access to obstetric and family planning services.

**Key Implications:**

- Program implementing partners who are familiar with the local community and health care system can help foster an environment of collaboration, knowledge sharing, and coordination among stakeholders at all levels.
- Donors should consider providing flexible support that responds in real time to the needs identified through monitoring and evaluation data to achieve meaningful progress toward reducing maternal mortality.
- The Program's foundation for successful sustainability included: implementation by Tanzanian teams, as opposed to teams from the Global North, embedding all interventions within existing government health system structures to foster strengthening from within, and working in partnership with all levels of government and in service of government plans and priorities.

See related article by Dominico et al.

## INTRODUCTION

Despite a 38% decline in the global maternal mortality ratio (MMR) between 2000 and 2017, approximately 810 women continue to die every day from preventable causes related to pregnancy and childbirth. Sustainable Development Goal (SDG) 3 includes a target to reduce the global MMR by 2030 to less than 70 maternal deaths per 100,000 births.^1^ With the most recent global estimate of MMR being 211 maternal deaths per 100,000 births;^2^ achieving the ambitious SDG target will require determined effort to implement and sustain high-quality obstetric and reproductive health services, particularly for the most underserved and rural communities. Business as usual simply will not do.

The Program to Reduce Maternal Deaths in Tanzania (hereafter referred to as the “Program”) was a 13-year effort (2006–2019) in the Kigoma region. At the time of its initiation, the MMR in Tanzania was 578 per 100,000 live births,^3^ and Kigoma was among the regions with the poorest maternal health indicators. Yet by 2019, there was marked improvement across a number of these indicators in Kigoma, and in some cases, the progress made surpassed national averages.

The approach taken was unique and adaptive. This was not a short-term, donor-driven pilot project with a rigid, predetermined set of interventions designed by and managed from the Global North but an organically developed initiative designed to fit the health conditions and needs of the region. This experience revealed that it is possible to significantly improve maternal and reproductive health in remote, low-resource settings, but this requires several factors: long-term investment; robust, multipartner collaboration in which the government plays a central role; locally determined and context-appropriate interventions; and demand creation that rests on a foundation of well-functioning and high-quality clinical services. Also essential is a willingness to not just collect evaluation data, but to respond to it, which may mean deviating from initial plans and strategies. We describe the Program's key interventions, how these evolved over time, major outcomes, and lessons learned. We hope this experience is instructive to others working toward maternal mortality reduction.

The Program was an organically developed initiative designed to fit the health conditions and needs of Kigoma.

## PROGRAM TO REDUCE MATERNAL DEATHS IN TANZANIA

The Program started in late 2006. At the time, approximately 12,000 women died annually in Tanzania as a result of pregnancy and childbirth, which placed it sixth in the world for maternal deaths (tied with Pakistan and Indonesia).^2^ Maternal mortality in Kigoma was one of the highest in the country.^4^ The direct causes of maternal death in Tanzania were primarily obstetric hemorrhage, sepsis, and hypertensive disorders.^5^ Contributing factors included: poverty; delayed health care seeking; a large rural population for which specialized, lifesaving care requires traveling long distances; high fertility and high unmet need for contraceptives; a shortage of skilled health care providers; and poor quality of care at health facilities.^6^^–^^8^ Further, the region lacked actionable, accurate, and timely information that could be used to plan and monitor interventions.

Although the high number of maternal deaths in Tanzania was a compelling justification for action, it was a novel and locally devised approach that provided the impetus for the Program. Dr. Godfrey Mbaruku, a Tanzanian obstetrician who served as a regional medical officer in Kigoma, proposed a strategy to decentralize lifesaving emergency obstetric care, traditionally offered in hospitals, to health centers that women could more readily access. Given that an estimated 15% of pregnant women experience obstetric complications requiring emergency care,^9^ and since such complications are hard to predict, emergency obstetric care must be accessible to **all** women at **all** times.

Dr. Godfrey Mbaruku proposed a novel and locally devised approach to address high maternal mortality in Tanzania: decentralize lifesaving comprehensive emergency obstetric care to health centers.

Recognizing that few doctors and no obstetricians worked within the public health care system in rural communities in Kigoma, and based on evidence from other countries,^10^^,^^11^ Dr. Mbaruku leveraged Tanzania's use of task sharing, which allowed certain cadres of associate clinicians—such as assistant medical officers (AMOs)—to provide comprehensive emergency obstetric and newborn care (CEmONC). Tanzania is one of the few countries in Africa where AMOs can perform cesarean deliveries and other obstetric surgeries and nurses and clinical officers provide anesthesia.^12^

AMOs have been trained and deployed in Tanzania since 1963 and are well-accepted and recognized among physicians.^13^ These health providers were more likely than medical doctors to live and work in rural areas and to perform most obstetric surgeries.^14^ The quality of surgical care in Tanzania by AMOs and medical doctors was similar,^13^ and both groups reported similar training experiences and post-training challenges.^15^ In addition to task sharing, Dr. Mbaruku recommended the simultaneous upgrade of existing, mostly rural, health centers with operating theaters, critical renovations, equipment, and supplies. This meant that health providers could perform all critical EmONC services on location, rather than referring women with life-threatening complications to hospitals several hours away.

This 2-pronged approach—task sharing and infrastructure upgrades—formed the basis of the initial investment by Bloomberg Philanthropies in 2006. The approach was subsequently adopted in the Government of Tanzania's policy, “The National Road Map Strategic Plan to Accelerate Reduction of Maternal, Newborn and Child Deaths in Tanzania 2008–2015—the One Plan.”^16^ The One Plan established an ambitious MMR goal to be achieved through the following operational targets: increasing institutional delivery to 80%; increasing modern contraceptive prevalence to 60%; ensuring all hospitals provide CEmONC; upgrading 50% of health centers to provide CEmONC; and increasing the number of facilities that provide basic EmONC to 70%.^16^ The One Plan, and subsequent updates to the national strategy, served as the blueprint for all Program activities.

Initially, the Program operated in underserved communities and health facilities in 3 regions of Tanzania (Kigoma: 6 health centers and 3 hospitals; Morogoro: 3 health centers and 1 hospital; and Pwani: 1 health center and 1 hospital). These communities and facilities were selected at the direction of the Tanzanian government, with the vast majority of investment in Kigoma. Support for Morogoro and Pwani ended in December 2015. The government recommended winding down support in these regions—both more urban, less disadvantaged, and with high facility delivery rates, 75% and 83%, respectively^17^—and to focus support in Kigoma. The government's recently issued “Sharpened One Plan” underscored prioritizing the country's Lake and Western Zones, to which Kigoma belongs, as these areas had the highest maternal and newborn mortality.^18^ The consolidation of resources—both human and financial—in a particularly needy region allowed the Program to cover more of the region and implement a more comprehensive set of interventions. Support for Kigoma continued through April 2019, when the Program's aims were achieved. We focus on the experience in Kigoma because it was the most comprehensive effort and important progress was made at a relatively large population scale, thus providing critical lessons for other regions grappling with high maternal mortality.

We focus on Kigoma because it was the most comprehensive Program effort and important progress was made at a relatively large population scale.

### Program Setting

Kigoma is 1 of 31 administrative regions in Tanzania. It is situated in the northwest of the country along the eastern shore of Lake Tanganyika. Kigoma region has a population of 2.3 million (5% of Tanzania's total population in 2015). In 2004, Kigoma lagged behind the national average for delivery in a health facility, cesarean delivery, and unmet need for family planning (FP), among other indicators (Table 1). Kigoma also had an extreme shortage of skilled health providers. In 2013, the density of skilled birth attendants (4.6 per 10,000 population)^19^ was nearly 10 times lower than the minimum recommended by the World Health Organization (44.5 per 10,000 population).^20^

**TABLE 1.** Key Maternal and Reproductive Health Population-Based Indicators for Tanzania (2004, 2010 and 2015/16), Western Zone (2004, 2010, 2015/16), and Kigoma Region (2004, 2010, 2014, 2015/6 and 2018), Women Aged 15–49 Years

	Tanzania			Western Zone^a^			Kigoma Region^b^				
	2004^d^	2010^e^	2015/16^f^	2004^d^	2010^e^	2015/16^f^	2004^d^	2010^e^	2014^g^	2015/16^g^	2018^h^
Population^c^	34,443,603	42,360,831	48,283,107	4,633,738	5,635,918	6,398,886	1,674,047	2,028,202	2,179,000	2,339,684	2,453,336
% Urban (women aged 15–49 years)	28.4	28.5	36.3	N/A	N/A	N/A	N/A	N/A	19.7	N/A	24.0
% Literate (women aged 15–49 years)	67.3	72.2	76.8	55.2	62.0	65.1	64.8	71.7	69.1	69.0	69.4
Total fertility rate (births per woman)	5.7	5.4	5.2	7.3	7.1	6.7	N/A	N/A	6.7	N/A	6.3
Currently using any method of contraception (women in union)	26.4%	34.4%	38.4%	12.8%	20.1%	22.8%	19.8%	25.2%	20.6%	24.1%	26.3%
Currently using modern contraception (women in union)^i^	20.0%	27.4%	32.5%	8.7%	14.6%	19.3%	12.2%	14.4%	15.6%	17.5%	21.0%
Attended at least 4 antenatal care visits	61.5%	42.8%	50.6%	N/A	N/A	N/A	N/A	N/A	42.1%	N/A	57.7%
Delivered in a health facility	47.1%	50.2%	62.6%	45.5%	36.5%	49.7%	39%	33.3%	47.1%	46.1%	77.0%
Delivered by cesarean section	3.2%	4.5%	5.9%	1.8%	2.8%	3.2%	1.9%	2.0%	3.5%	4.0%	5.2%
Perinatal mortality rate (per 1,000 births)^j^	42	36	39	28	29	32	N/A	N/A	29	N/A	32
Maternal mortality ratio (per 100,000 live births)	578	454	556	N/A	N/A	N/A	N/A	N/A	N/A	N/A	N/A
Under-5 mortality rate (per 1,000 live births)	112	81	67	138	98	69	N/A	N/A	56	N/A	48

Abbreviation: N/A, data not available.

a Includes Tabora, Shinyanga, and Kigoma regions.

b Regional estimates from national Demographic and Health Surveys conducted in 2004, 2010, and 2015/16 are based on small samples (200–500 women); maternal mortality at subnational level not available due to small sample sizes.

c Population projections based on the 2002 and 2012 census rounds.^21^

d Source: 2004 Tanzania Demographic and Health Survey.^3^

e Source: 2010 Tanzania Demographic and Health Survey.^22^

f Source: 2015–2016 Tanzania Demographic and Health Survey.^17^

g Source: 2014 Kigoma Reproductive Health Survey.^23^

h Source: 2018 Kigoma Reproductive Health Survey.^24^

i Modern contraception includes male or female sterilization, intrauterine contraceptive devices, injectables, implants, pills, male or female condoms, diaphragms, foam or jelly, lactational amenorrhea method, and emergency contraception.

j Stillbirths and early neonatal deaths that occurred in the 5 years before the survey per 1,000 total births (stillbirths and live births).

## PROGRAM APPROACH

**FIGURE 1:**
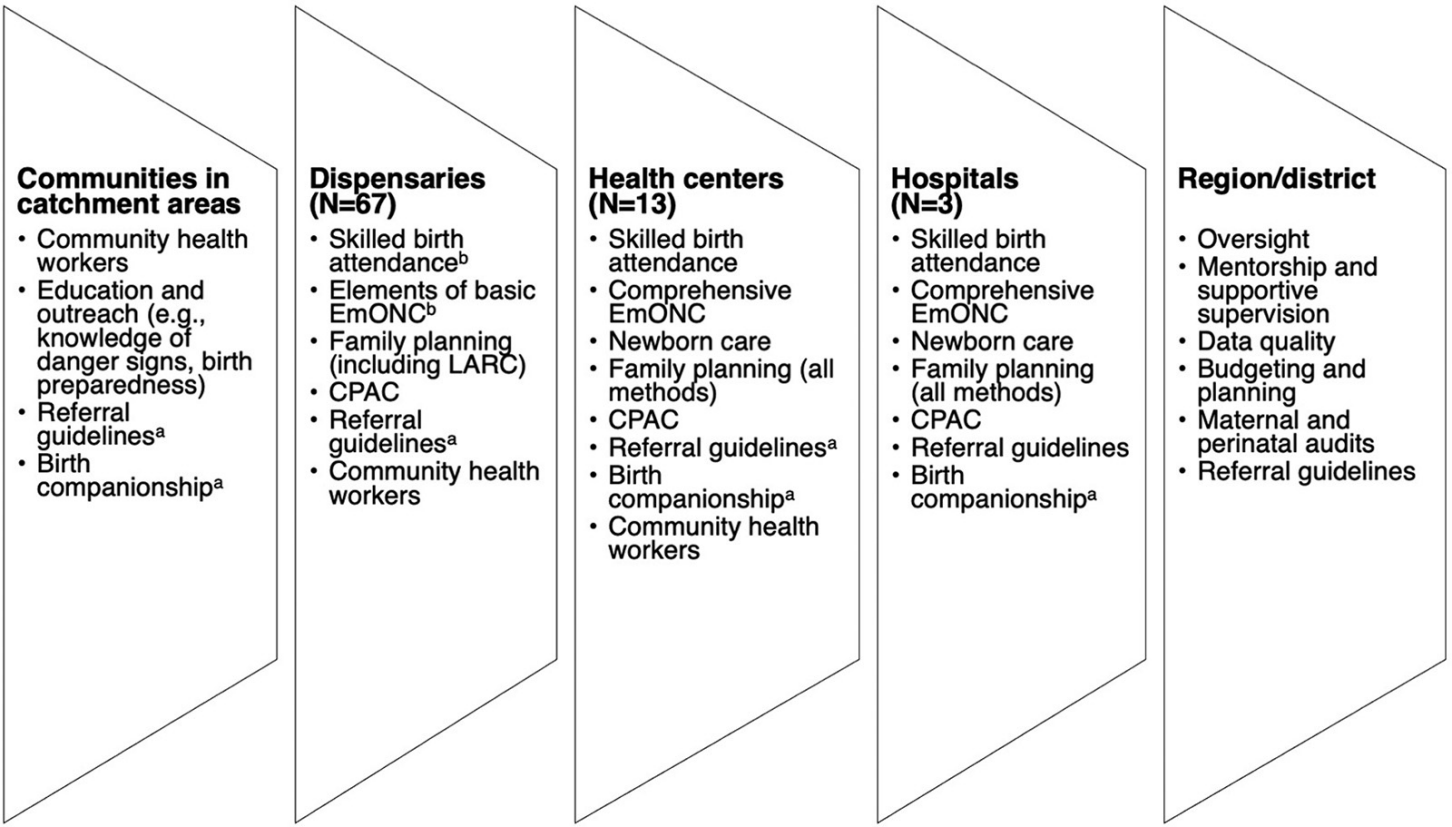
Program Logic Model Abbreviations: GoT, Government of Tanzania; EmONC, emergency obstetric and newborn care; MRH, maternal and reproductive health; MMR, maternal mortality ratio. ^a^Partnership is GoT, communities, health facilities/providers, donors, implementing partners, and evaluator. ^b^MRH services is EmONC, skilled birth attendance, newborn care, family planning, and comprehensive postabortion care. ^c^Quality in terms of clinical services and women's experience of care.

The initial conception of the Program and what it ultimately became are significantly different. We provide an overview of the Program's interventions in Kigoma and how and why the program evolved over time. Greater detail about each element of the program is included elsewhere.^19^^,^^25^^–^^28^ An overview of the Program's enabling conditions, strategies, outcomes, and impacts are presented in a logic model in Figure 1.

### Enabling Conditions

A critical feature of the Program was the multipartner collaboration, which recognized that each partner had a unique role, and by joining efforts, it was possible to achieve much more. The Government of Tanzania participated at multiple levels: nationally through the Ministry of Health, Community Development, Gender, Elderly, and Children (MOHCDGEC) and the President's Office, Regional Administration and Local Government; regionally through the health management team; and at the district level via council health management teams. Rounding out the core partnership were implementing organizations with expertise in obstetric care and reproductive health care (Thamini Uhai/Vital Strategies and EngenderHealth, respectively); an evaluation partner (U.S. Centers for Disease Control and Prevention); and donors (with major funding from Bloomberg Philanthropies and Fondation H&B Agerup).

The partnership met regularly to review progress and plans, including monthly meetings convened by the Regional Medical Officer. The Program had a very strong stakeholder engagement strategy; all key stakeholders, including the health providers and the community, were regularly consulted before any new intervention was implemented. All meetings to disseminate evaluation findings were attended by government officials and health providers, and their feedback informed Program improvements. It is worth noting that when the decision to focus on Kigoma was made, both implementing organizations established offices there and relocated staff, and Bloomberg Philanthropies brought on a Tanzanian program manager to coordinate the effort from Kigoma. The presence of partners locally allowed for constant communication and collaboration with local government and health facility staff, a deeper understanding of the local needs and context, and the building of trust with local communities. Furthermore, the Program was able to form an administrative and technical structure that, over time, built the capacity of the regional and council health management teams (R/CHMTs) to provide operational, technical, and logistical support to promote sustainability.

The presence of partners locally allowed for constant communication and collaboration with local government and health facility staff, a deeper understanding of the local needs and context, and the building of trust with local communities.

Total donor support for the Program, across all regions over 13 years, was approximately US$44 million. The vast majority of funding went to implementation activities and partners in Tanzania. While Bloomberg Philanthropies and Fondation H&B Agerup contributed most of the funds, the Program also received funding for discrete programmatic components from Blue Lantern Foundation, Merck for Mothers, Svenska PostkodStiftelsen, and the Swedish International Development Cooperation Agency (Sida). The Program operated within the public health care system, and as such, additional costs were borne by the Tanzanian government (e.g., health provider salaries, routine operational costs, materials, and supplies). A detailed cost analysis is not included in this article; the external funding amount is provided to give donors and implementing organizations an idea of what it costs to support an initiative of this nature.

### Program Strategies and Interventions

**FIGURE 2:**
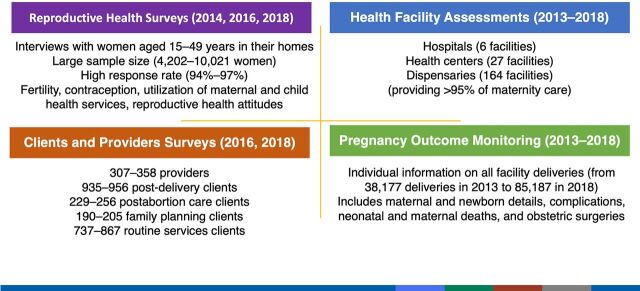
Snapshot of Major Program-Supported Interventions by Level of Health System Abbreviations: CPAC, comprehensive postabortion care; EmONC, emergency obstetric and newborn care; LARC, long-acting reversible contraceptive. ^a^In select catchment areas/facilities. ^b^In a subset of 18 dispensaries.

The Program evolved over 3 phases: Phase 1 (2006–2012), Phase 2 (2012–2015), and Phase 3 (2015–2019). Within each phase, the interventions implemented can be categorized into 3 broad strategies: (1) to increase and sustain the **availability** of high-quality maternal and reproductive health services; (2) to improve and sustain **access** to maternal and reproductive health services; and (3) to create and sustain **demand** for maternal and reproductive health services (Table 2). These interventions were implemented in and around the catchment areas of the supported health facilities (Figure 2). By the end, the Program supported 83 health facilities in Kigoma (3 hospitals, 13 health centers, and 67 dispensaries).

**TABLE 2.** Strategies and Interventions by Phase and Level of Implementation (Activities Conducted by Program Unless Otherwise Noted)

	Phase 1 (2006–2012)	Phase 2 (2012–2015)	Phase 3 (2015–2019)
Increase and sustain availability of high-quality MRH services			
RHMT/CHMTs	-Reviewed and approved all activities and materials (e.g., job aids, campaign content) -Participated in supportive supervision and mentorship visits to facilities -Participated in CME workshops		
	The R/CHMTs were involved in all aspects of facility upgrades and planning provider training	-Improved FP commodities stock management and provided a buffer stock of all commodities in case of stock-outs -Provided training of trainers in FP	-Formed a regional mentorship team -Provided training and technical support to improve quality of MRH data; use data for decision making, planning and budgeting; and conduct MPDSR -Supported to train district-based biomedical technicians to repair medical equipment -Participated in research studies (e.g., birth companionship, EmONC, and refugee communities) -Worked toward including additional MRH service delivery costs identified through the Program into budgets (e.g., cost of providing routine onsite supervision and mentorships visits)
Hospitals	(N=3) -Provided routine supportive supervision and mentorship -Conducted routine clinical audits -Participated in CME workshops		
	-Provided some EmONC equipment, supplies, and medications		Not applicable
	-Made minor renovations as needed -Provided training and supported health providers to deliver high-quality CEmONC -Built staff houses for maternity staff	-Provided training and supported health providers to deliver high-quality: CEmONC, LARC, PMs, and CPAC -Installed technical infrastructure and provided training in using e-learning system -Linked to toll-free closed user group mobile phone network for emergency calls and teleconferences	-Provided training and supported health providers to deliver high-quality CEmONC, LARC, PMs and CPAC, HBB,^a^ respectful maternity care -Provided training on improving quality of MRH data and using data for decision making, budgeting, and planning -Introduced birth companionship in 1 hospital
Health centers	(N=6) -Constructed 6 operating theaters and renovated maternity wards as needed -Provided EmONC equipment, supplies, and medications -Provided training and supported non-doctors to provide obstetric surgery and nurse-midwives/clinical officers to provide anesthesia -Provided monthly supportive supervision and mentorship visits -Conducted monthly clinical audits -Provided CME workshops as needed (e.g., on assisted vaginal delivery, infection prevention)	(N=12) -Constructed operating theaters in 5 additional health centers and renovated maternity wards as needed; provided technical assistance on upgrade of 1 additional health center -Provided EmONC & FP equipment, supplies, and medications -Provided training and supported health providers in CEmONC, SBA, LARC, PMs, and CPAC -Provided quarterly supportive supervision and mentorship visits -Conducted quarterly clinical audits -Provided CME workshops as needed -Linked to toll-free closed user group mobile phone network for emergency calls and teleconferences -Installed technical infrastructure and provided training in use of e-learning system -With a focus on improving quality of EmONC, introduced weekly teleconferences, emergency call system, and e-learning platform -Provided training and support to use COPE -Provided technical support for FP service days	(N=13) -Provided training and supported health providers in CEmONC, SBA, LARC, PMs, CPAC, use of simulations with mannequins to regularly refresh skills, respectful maternity care. -Provided quarterly supportive supervision and mentorship visits -Conducted quarterly clinical audits -Provided CME workshops as needed -Continued weekly teleconferences, emergency call system, and e-learning platform -Introduced HBB and KMC^b^ -Provided training and support to use COPE^c^ -Provided technical support for FP service days -Provided training to improve quality of MRH data and how to use data for decision making, budgeting, and planning -During critical shortages, provided essential EmONC equipment, supplies, and medications -Introduced birth companionship in 8 health centers
Dispensaries	Not applicable	Not applicable	(N=67) -Renovated 67 dispensaries -Provided equipment, supplies, and medications for some BEmONC, SBA, LARC, and CPAC -Provided training for health providers in BEmONC and skilled birth attendance (N=18) -LARC (N=67); and CPAC (N=35) -Provided monthly supportive supervision and mentorship visits, in partnership with closest health center in-charge -Provided training and support to use COPE -Provided CME workshops as needed -Provided technical support for FP service days
Improve and sustain access to MRH services			
RHMT/CHMTs	Not applicable	Participated in development and implementation of referral guidelines	
Hospitals	Not applicable	Participated in development and implementation of referral guidelines	
Health centers	Not applicable	Developed and implemented referral guidelines in partnership with catchment area around 1 health center	Developed and implemented referral guidelines in partnership with catchment areas around 3 health centers
Dispensaries	Not applicable	Developed referral guidelines in partnership with catchment areas around 5 dispensaries	Developed referral guidelines in partnership with catchment areas around 18 dispensaries
		-Provided technical support for FP outreach -Provided technical support for FP weeks	
Communities	Not applicable	-In partnership with health center and dispensaries in catchment area: developed referral guidelines; started emergency scheme funds; organized local transport providers to provide care to women during obstetric emergencies -Provided technical support for FP weeks -Integrated FP service delivery with other community events (e.g., immunization mobile teams)	
Create and sustain demand for MRH services			
RHMT/CHMTs	Not applicable	-Participated in the design and development of all multimedia communication campaigns to increase demand and utilization of services -Involved in the selection of 139 community members who were trained by the program as CHWs -Teamed up with program staff in conducting routine supportive supervision to CHWs	
Hospitals	Not applicable	Participated in campaigns	
Health centers	Not applicable	-Participated in campaigns -Provided capacity building and support to facility in-charges to supervise CHWs -Provided training to facilities to facilitate the process of community members being led in “walk throughs” to learn about services provided	
Dispensaries	Not applicable	-Participated in campaigns -Provided capacity building and support to facility in-charges to supervise CHWs	
Communities	Not applicable	-Exposed to 2 multimedia campaigns focusing on importance of facility delivery, birth preparedness, and FP	-Exposed to 1 multimedia campaign focusing on importance of facility delivery, birth preparedness, and birth companionship -Promoted the use of birth companions during facility deliveries
		-Supported CHWs to promote and educate women and communities on MRH -Provided support to CHWs to conduct outreach events -Provided support to cultural troops -Facilitated the collection of testimonies from satisfied clients -Worked with 2 CBOs to provide reproductive health education for adolescents in schools	

Abbreviations: BEmONC, basic emergency obstetric and newborn care; CBOs, community-based organizations; CEmONC, comprehensive emergency obstetric and newborn care; CHMT, council health management teams; CHWs, community health workers; CME, continuing medical education; COPE, client-oriented, provider-efficient; CPAC, comprehensive postabortion care; EmONC, emergency obstetric and newborn care; FP, family planning; HBB, helping babies breathe; KMC, kangaroo mother care; LARC, long-acting reversible contraceptive; MPDSR, maternal and perinatal death surveillance and response; MRH, maternal and reproductive health; PM, permanent methods; RHMT, regional health management team; SBA, skilled birth attendance.

a Helping Babies Breathe is a training curriculum designed to improve neonatal resuscitation skills through hands-on learning and practice using the NeoNatalie newborn simulator; the training was designed to specifically meet the needs of resource-limited settings.^37^

b Kangaroo mother care is a method of care initially designed for preterm and low birthweight infants that involves the infant being held to the mother's chest for skin-to-skin contact (usually in sessions of minimum 1 hour, several times per day), early exclusive breastfeeding, and early discharge from the health facility. It is initiated in health facilities by specially trained health care providers and can continue at home.^38^

c Client-oriented, provider-efficient is an approach that helps health care staff continuously improve the quality and efficiency of services provided at their facility and make services more responsive to clients' needs.^39^

**FIGURE 3:**
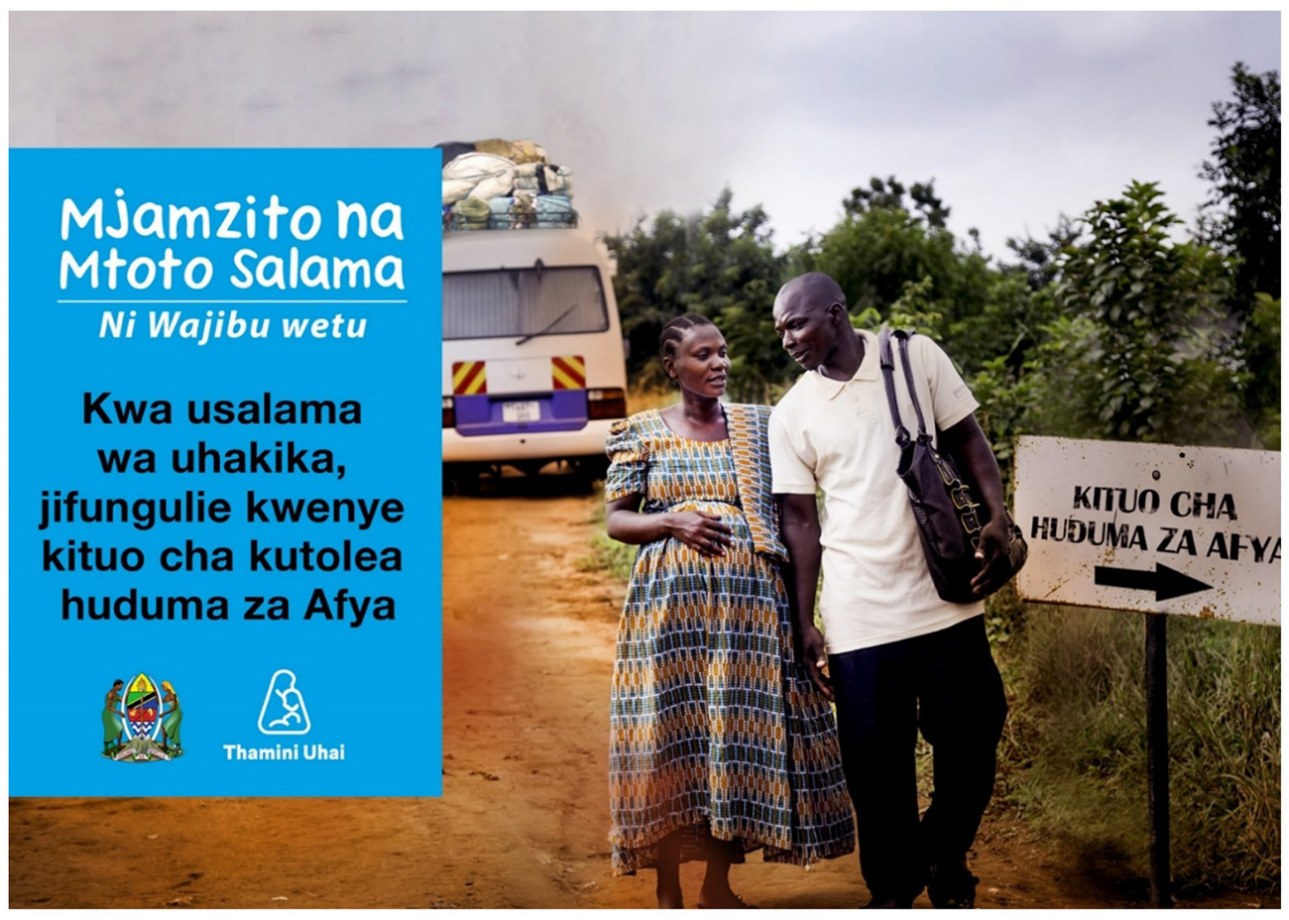
Evaluation Methods Source: CDC evaluation reports^23^^,^^24^^,^^29^^–^^36^^,^^40^

The phased approach allowed for gradual expansion of interventions and coverage. While each phase had distinct areas of focus, the program was nevertheless a continuum with later phases building on earlier phases. Cutting across the Program was the collection of evaluation data, including at the population level, where possible. These data served 2 related purposes: (1) to track progress; and (2) to shape the program's strategy through the identification of gaps. Phases 2 and 3 arose in response to evaluation findings that suggested changes and/or new interventions. The different types of evaluations that were conducted are described further in Figure 3 and published CDC evaluation reports.^23^^,^^24^^,^^29^^–^^36^^,^^40^

#### Phase 1

This phase largely focused on expanding the supply and quality of CEmONC, with an emphasis on expanding women's access to obstetric surgery. Specifically, 6 health centers were upgraded by constructing operating theaters and providing equipment and supplies. Three hospitals that served as referral sites for the upgraded health centers received critical renovations, supplies, and equipment. Housing was built for health providers as a recruitment and retention mechanism.

Simultaneously, associate clinicians and nurses underwent intensive 3-month competency training in CEmONC or anesthesia. The practical and theoretical curriculum was originally developed by the Ifakara Health Institute and endorsed by the MOHCDGEC.^25^ Once health providers completed their initial training, the focus shifted to improving the quality of care through the introduction of clinical audits, onsite supportive supervision and mentorship by experienced obstetricians from outside the region, and continuing medical education.^25^^,^^41^ All program activities were delivered in close coordination with the R/CHMTs, which helped solve problems using existing resources in the region/council or neighboring regions.

During the Program's first phase, availability of CEmONC was expanded by upgrading health centers.

First evaluation efforts (2010–2011) included a mixed-method approach that documented the operational performance and outcomes in program-supported facilities.^42^ Service statistics and an assessment tool were used to evaluate the functionality of infrastructure and availability of EmONC signal functions and essential services. In-depth interviews were conducted with health care workers, district health officials, and program staff to gain an understanding of their experiences and perceptions of the program and gather insights on facilitators and barriers to implementation.

#### Phase 2

This phase was marked by 3 major programmatic enhancements. The first was more robust, systematic, facility- and population-level evaluation data to gauge impact (Figure 1 and Table 2). Evaluation methods used during Phase 1 were fundamental for program design but did not provide an adequate baseline that could be used to measure progress and determine the impact on maternal mortality. At the request of the regional and national government, the evaluation design changed in 2013 to document functionality and outcomes in all health facilities (governmental and private), providing care for more than 95% of facility-based deliveries in Kigoma.^30^^,^^32^ Health facility assessments and pregnancy outcomes monitoring using individual clinical data were conducted every other year to capture provision, quality, and utilization of services, and their coverage and impact.^31^^,^^34^ Facility monitoring was complemented by large-scale, population-based surveys of women ages 15–49 years starting in 2014 and repeated in 2016 and 2018 to capture changes in fertility, contraceptive use, service utilization, and other health behaviors.^23^^,^^24^ Results from all surveys were disseminated at workshops with regional and district officials, health providers, and other nongovernmental organizations working in Kigoma.

**Figure:**
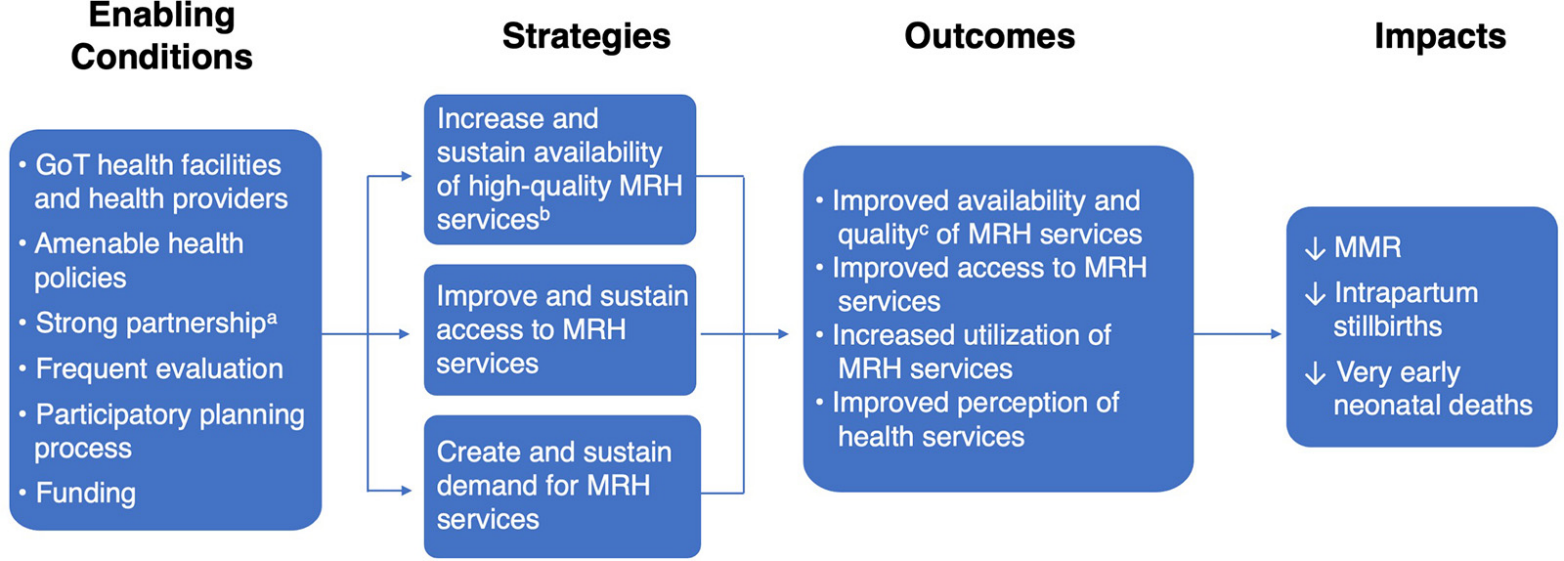
A family planning campaign poster shows a supportive partner escorting a pregnant woman at term to a health facility for delivery and states: “For safety and reliability, please deliver in a health care facility.” © 2018 ABC Bros Co Ltd.

The second programmatic enhancement emerged through a new funding commitment made by Bloomberg Philanthropies to support FP globally. Incorporating FP services was logical given the large unmet need for modern contraceptives and the estimate that eliminating this need could reduce the annual global number of maternal deaths by an additional 27%.^43^ The Program supported FP outreach education and distribution services (e.g., FP weeks, service days, integrated FP service delivery) in communities; it also strengthened the provision of high-quality FP services by working with the R/CHMTs to integrate FP into routine services and to expand method choices. This approach ensured that the Program was able to increase demand for FP services while meeting the need of more clients in multiple locations. The Program also strengthened the R/CHMT's overall capacity to manage FP services through training-of-trainers for each district and by centralizing FP-related training and mentorship for health facilities. In addition, a large burden of maternal mortality and morbidity from complications of abortion necessitated the inclusion of comprehensive postabortion care (CPAC) within the Program.

Integrating FP and postabortion care services into the Program was logical given the large unmet need for modern contraceptives and that reducing this need could help reduce maternal mortality.

All 6 health centers supported by the Program received minor renovations as well as equipment and supplies, and health providers across various cadres were trained to provide FP and CPAC. Seven additional health centers were selected and supported during Phase 2, with upgrades and training for the full package of services: CEmONC, FP, and CPAC. Efforts to further improve and to maintain quality of care through supportive supervision and clinical audits continued and were augmented by using the client-oriented, provider-efficient approach;^39^ specialized workshops; the installation of an e-learning platform; and a 24-hour emergency call system that allowed providers to reach experienced obstetricians for clinical support.^44^ These additional facilities were selected based on geographic analysis of the distance between communities and existing EmONC services, population coverage, and existing infrastructure and human resources. All decisions were made in close collaboration with local and regional government authorities.

The third programmatic enhancement focused on building demand for health services. Findings from the 2013 health facility assessment and pregnancy outcomes evaluations revealed that, while access to CEmONC services had improved, key indicators remained low, including the population-level institutional delivery rate (48.8%), met need for obstetric care (44%), and cesarean delivery rate (2.6%).^30^ This was addressed through an evidence-based, multiplatform communication campaign that highlighted the importance of facility delivery, birth planning, and pregnancy danger signs.^45^ An additional multimedia communication campaign on FP was also launched to increase demand.^46^ Community health workers were engaged, serving as trusted resources and liaisons between communities and the health care system.

#### Phase 3

This phase coincided with the decision to wind down efforts in Morogoro and Pwani and ramp up efforts in the Kigoma region. At this point, the Program established operational targets to improve the institutional delivery rate, met need for emergency obstetric care, and the modern contraceptive prevalence rate, all aligned with government targets.

The Program adopted the government's operational targets to improve institutional delivery rate, met need for EmONC, and modern contraceptive prevalence rate.

Accordingly, this phase was marked by 4 programmatic enhancements that had several benefits: (1) an expanded geographic reach, covering almost all of Kigoma's 2 million inhabitants; (2) strengthened referral systems; (3) improved newborn health outcomes, which had not been an explicit focus for the Program but lagging indicators compelled action; and (4) a person-centered, improved health care experience, bolstering demand for services. At the same time, the Program continued to support demand creation with a third mass-media campaign and further support for community health workers. Reaching young people was a challenge, so the team partnered with a local youth-led organization to provide FP information and to mobilize young people to access FP and CPAC services in supported health facilities; efforts were made to make facilities more youth-friendly. Community-based activities were also supported by the mass media campaign, and health education sessions were used to dispel myths and misconceptions about services. The evaluations initiated in Phase 2 continued and new evaluations were introduced in Phase 3 (described further later). The process of phasing out direct Program support and handing over the Program to the government was integrated into all aspects of Phase 3 and included building the capacity of the R/CHMTs to provide facilities with mentorship and in-service training.

**Decentralize Emergency Obstetric Care, FP, and CPAC.** The first program enhancement was further decentralization of emergency obstetric care, FP (particularly provision of long-acting contraceptives to expand women's method choice), and CPAC to dispensaries, which are much more numerous and accessible. CDC evaluations showed that dispensaries were the most used level of facility for preventive services and delivery care. These findings aligned with the government's national strategy to improve maternal and reproductive care in primary health facilities. In total, 67 dispensaries were supported through infrastructure upgrades, training, and supportive supervision. All 67 were upgraded to provide FP, of which 35 were supported to provide CPAC; within this group, 18 were supported to provide some of the lifesaving interventions required for basic EmONC (primarily injectable antibiotics, uterotonics, anticonvulsants, removal of retained products, and neonatal resuscitation). The same criteria used for the selection of health centers in Phase 2 were used to select dispensaries.

**Strengthen Referral Systems.** While strength-ening service delivery at the dispensary level further expanded women's geographic access, it was also important to strengthen referral systems for obstetric complications. For the Program's second enhancement during this phase, the team worked with communities, providers, and government officials to design and enable context-specific referral pathways to reduce delays in women reaching EmONC services. The focus was on developing local solutions: creating detailed referral plans in communities and facilities, establishing community-managed funds to cover the cost of transport (Box), and partnering with community-based transport providers to supplement/fill in gaps in ambulance coverage.

BOX **Community-Managed Fund for Referral**

When women experience obstetric emergencies, delays can be deadly. In Kigoma, community emergency funds, established with the help of Thamini Uhai's referral program, removed a critical barrier—money to transport women to emergency care.

The value of the funds, which the communities managed, became evident to Shamira (not her real name), age 24 years. The mother of 3 was in labor with her fourth child at the Gwarama dispensary near her home. Her health provider realized her labor was not progressing and decided to refer her to Nyanzige health center in Kakonko district, one of the upgraded health centers offering comprehensive emergency obstetric and newborn care (CEmONC).

The problem was transport. Nyanzige is 15 km from Gwarama, and the catchment area's only ambulance was not working.

*I was scared and thought I was going to lose my pregnancy or die because there was no possibility of getting the ambulance. We had no money in the house and my husband didn't contribute to the emergency fund when the village leaders asked him to contribute.* —Shamira

While everyone in the community was asked to participate in the fund, the funds were available to all families, including those who were unable to contribute.

Her health provider went to the village chairman who asked the emergency fund committee to release the funds to cover the cost of hiring private transport. At the time of Shamira's obstetric emergency, the fund in Gwarama had more than 247,000 TZS (about US$95) in reserve, of which 25,000 TZS was used to transport her to the CEmONC center at Nyanzige.

The *bodaboda* driver (motorcycle) arrived and quickly brought Shamira to Nyanzige health center, where she had a cesarean delivery.

*The community emergency scheme fund saved my life and the baby. Without it, I could have died.* —Shamira

**Improve Newborn Health Outcomes.** To complement the extensive work done to improve the quality of intrapartum services for women, and to respond to evaluation results showing persistently high perinatal deaths, the Program's third enhancement during this period was focused on newborn care. This included introducing Helping Babies Breathe^37^ in supported facilities to improve the skills of health providers to resuscitate newborns with asphyxia using national training approaches and guidelines.^47^ Similarly, the Program organized Kangaroo Mother Care^38^ wards/corners for preterm and low birthweight babies in supported facilities, an approach endorsed by national guidelines^48^ but only recently scaled up country-wide and included in the national strategy.^49^

**Improve the Health Care Experience.** Over time, as the technical/clinical quality of services improved and the Program looked to attract more women to facilities, it began to focus on strengthening the nonclinical elements of quality of care (i.e., experience of care). This new area of work, the fourth enhancement, was informed by a new evaluation method: interviews with health providers and with women being discharged (Figure 3).^35^^,^^36^ Findings led to the inclusion of respectful maternity care into continuing medical education for health providers in supported facilities and to the development and pilot of Tanzania's first birth companionship project, which enabled women to have continuous emotional, informational, and practical support during childbirth in select facilities. The positive findings from the pilot^50^ resulted in birth companionship being promoted as standard practice in Tanzania through its inclusion in the “National Guidelines for Gender and Respectful Care Mainstreaming and Integration Across RMNCAH services in Tanzania.”^51^

In 2015, it was decided that financial and technical support for the Program would conclude in 2019, assuming it continued its trajectory of progress and that a scalable model that the government could adopt was established. By early 2017, it was evident that the Program was on track to meet or surpass many of the programmatic targets set out in the government's “Sharpened One Plan”^18^ and in consultation with government officials, the decision was made to focus on transitioning full oversight of the Program to the government. Efforts to promote program sustainability were intensified. Objectives were twofold: (1) to enable continuity of high-quality care; and (2) to urge the Government of Tanzania to allocate sufficient budget to continue, and ideally scale, the Program's interventions. For the next 2 years, partners mostly moved from direct implementation of Program activities to supporting efforts to achieve both sustainability objectives. The Program brought on the Global Health Advocacy Incubator to support the development and implementation of a roadmap for financial and technical sustainability.

By 2017, it was evident that the Program was on track to meet or surpass many of the programmatic targets set out in the government's “Sharpened One Plan.”

For the first objective, the team built the capacity of Kigoma-based stakeholders to maintain all aspects of service delivery, notably by establishing a multidisciplinary, regional mentorship team that continued onsite supportive supervision and mentorship in program-supported facilities. In addition, health facility managers received training for administrative and operational tasks, and technicians were trained to maintain and repair equipment.

Efforts to achieve the second objective, ensuring adequate financial resources for Program continuity, consisted of direct budget and planning support to district councils, as well as garnering high-level political support. With respect to the former, health facilities are tasked with projecting their annual budgets, which feed into district council budgets and plans; these, in turn, are submitted to the national government for approval and disbursement. Program partners developed a list of priority activities with associated costs and worked with facilities, district councils, and the Kigoma regional health team to incorporate these costs into their annual budget requests. This process was supported for 2 budget cycles. In addition, a forum with high-level district and regional officials was held to ensure that those in a position to draft, influence, approve, and advocate for increased budget allocations had the capacity to do so effectively.

In parallel, an advocacy campaign directed at national-level decision makers was undertaken to raise the profile of the Program's impact, positioning it as a national model and advocating for increased and sustained funding. A series of meetings were held with key decision makers in critical ministries to make the case for increased budget allocations; these engagements allowed the team to identify high-level champions. In addition, journalist trainings, editor roundtables, and press conferences generated media stories on the issue of maternal mortality and the Program's impact in Kigoma, which played an important role in drawing national attention among both the population and policy makers. This resulted in regional and national level commitments to sustaining good-quality service delivery.

## PROGRAM OUTCOMES AND IMPACT

By the end of the Program, the evaluation revealed a 43% reduction in the facility-based maternal mortality ratio (MMR) in Kigoma, from 303 to 174 per 100,000 live births (Table 3). The institutional intrapartum stillbirth rate declined by 58% (from 14.4 to 6 intrapartum stillbirths per 1,000 births) and the predischarge neonatal mortality rate fell by 29% (from 10.7 to 7.6 per 1,000 live births). All declines were statistically significant. While inputs were facilitated by the Program in 83 supported health facilities (of 197 facilities providing maternity care), and Program-supported facilities had better outcomes overall, interventions were codesigned and implemented with local stakeholders, and elements of the approach were replicated in nonsupported facilities by regional and district health planners and managers. This is likely to have contributed to region-wide improvements.

**TABLE 3.** Selected Facility and Population-Based Indicators Documented by External Monitoring, Kigoma Region, 2013/2014 and 2018^a,b^

Indicators From Facility-Based Surveys	2013	2018	% Change	Significance^c^
Institutional maternal mortality ratio (per 100,000 live births)	303	174	-43	***
Predischarge neonatal mortality rate (per 1,000 live births)	10.7	7.6	-29	***
Institutional intrapartum stillbirth rate (per 1,000 births)	14.4	6.0	-58	***
Number of BEmONC facilities^d^	2	6	+200	NA
Number of CEmONC facilities^d^	9	15	+67	NA
**Indicators from population-based surveys (RHS)**	**2014**	**2018**	**% Change**	**Significance** ^c^
Contraceptive prevalence all methods (current use among married women aged 15–49 years)	20.6	26.3	+28	***
Contraceptive prevalence modern methods (current use among married women aged 15–49)	15.6	21.0	+35	***
Prevalence of implant and IUD use (current use among married women aged 15–49 years)	2.1	9.4	+348	***
Unmet need for contraception (married women aged 15–49 years)	39.2	35.1	-11	***

Abbreviations: BEmONC, basic emergency obstetric and newborn care; CEmONC, comprehensive emergency obstetric and newborn care; EmONC, emergency obstetric and newborn care; IUD, intrauterine device; RHS,, reproductive health survey.

a Source: CDC evaluations in health facilities^30^^,^^31^ and population-based surveys.^23^^,^^24^^,^^29^

b Baseline/endline population-based indicators were measured in mid-2014 (2014 RHS)^23^ and mid-2018 (2018 RHS).^24^

c Asterisks indicate significance level of the difference between baseline and endline outcomes for all facilities combined, using a z-statistic test for rates and proportions to calculate the p-value of the difference, as follows: *** = *P*<.01. NA=Not applicable.

d Include facilities with provision of lifesaving interventions that constitute EmONC that performed these interventions in the last 3 months: BEmONC interventions include administration of parenteral antibiotics, uterotonics, or anticonvulsants; manual removal of placenta; removal of retained products; assisted vaginal delivery; and basic neonatal resuscitation. CEmONC interventions include 2 additional services: ability to perform obstetric surgery (e.g., C- section) and blood transfusion; BEmONC and CEmONC facilities may or may not have performed assisted vaginal delivery in past 3 months (i.e., BEmONC-1 and CEmONC-1). According to the World Health Organization—which recommends at least 5 EmONC facilities per 500,000 population, including at least 1 CEmONC facility—by 2018 Kigoma achieved a sufficient number of CEmONC facilities but is still lagging behind in BEmONC facilities.^9^

These favorable maternal and neonatal outcomes were complemented by significant increases in current contraceptive use—particularly the use of long-acting, reversible contraceptives and other modern methods among married women, which rose by 35% (from 15.6% to 21.0%). Consequently, the unmet need for contraception declined by 11% (from 39.2% to 35.1%).

Favorable maternal and neonatal outcomes were complemented by significant increases in current contraceptive use.

## LESSONS LEARNED

Our experience in Kigoma demonstrates that decentralizing high-quality CEmONC, FP, and CPAC in remote, low-resource settings is both feasible and effective and can be considered in places with similar contexts. Here we describe 4 critical lessons learned for governments, donors, and implementing organizations working to reduce maternal mortality.

### 1. Multistakeholder Partnerships Are Critical

First, multistakeholder partnerships are critical. Where resources are limited, no one stakeholder can effectively address a complex problem like maternal mortality in a high-quality, comprehensive, and sustainable manner. A major strength of the Program was the collaboration between all levels of government, implementing partners, evaluation partners, funders, health providers, and the communities of Kigoma. These relationships deepened over more than a decade and were built around a shared commitment to improving maternal and newborn health. The government played a central role in setting the strategy, and all partners were committed to keeping Program interventions within the confines of the public health care system, thus avoiding the creation of unsustainable parallel systems.^52^ Partners came together regularly to develop integrated workplans across organizations, assess progress, and plan adjustments, ensuring that everyone was on board and “owned” the ongoing implementation.

*[The Program] approach has worked because they went to the people and came up with what the people actually wanted before implementing the project…. We will sustain the achievements.*^52^ —Dr. Leonard Subi, Director of Preventative Services, MOHCDGEC

A major strength of the Program was the collaboration between all levels of government, implementing partners, evaluation partners, funders, health providers, and the communities of Kigoma.

In the last 4 years of the Program, Bloomberg Philanthropies brought on a Tanzanian program manager who was based in Kigoma and fostered an environment of collaboration, knowledge sharing, and coordination. In retrospect, it would have been advantageous to bring on such a program manager from the start.

### 2. Increasing Both Supply and Demand of Services Is Important

A second lesson learned is the importance of increasing both the supply of high-quality maternal and reproductive health services and the demand for these services. Initially, the Program was focused on increasing the number of health facilities capable of providing EmONC, based on the assumption that better availability of high-quality care would result in increased use of services. Yet, the 2013 pregnancy outcomes evaluation showed this was not the case. It was at this point that the Program began its demand-creation activities—multilevel communication campaigns and the recruitment and support of community health workers to serve as trusted sources of information. Two critical factors that allowed the Program to expand in this way were the flexibility of the funders to make resources available for activities that were not initially anticipated and the engagement of technical experts to design locally resonant and evidence-based communications campaigns. Demand creation must rest on a foundation of well-functioning and high-quality clinical services if it is to be effective—driving people to services that are not available or effective can backfire—and demand-side activities need to be anticipated from the start of a program and implemented as soon as quality services are available.

### 3. Respond in Real Time to Monitoring and Evaluation Data

The third lesson is the importance of not only collecting robust monitoring and evaluation data but also being responsive in real time to what the data reveal. For example, the Program did not initially plan to carry out demand-generation activities, but these activities were incorporated based on interim evaluation data. It was also important to monitor the functioning of services over time, as service readiness (e.g., transfer of health providers trained by the Program to other facilities and broken equipment) fluctuated.^19^ This has 2 implications: first, the need to conduct (and therefore, fund) evaluations at regular intervals over the life of a program, not only at the beginning and the end; and second, the need for donor support to be flexible and potentially long term. In Kigoma, donors did not anticipate at the outset that support for the Program would continue for 13 years; however, as evaluation data revealed the need for course corrections and additional interventions to better serve the local population, donors felt compelled to keep going until a model was realized that the government could maintain and ideally scale. Complex, multifaceted issues such as maternal mortality do not lend themselves to quick fixes. While some may argue that the resources required to conduct rigorous, population-level evaluations displace resources for service delivery, our experience showed that the former was critical to optimizing the latter. A readiness among donors to provide flexible support that responds to the needs identified through monitoring and evaluation will go a long way toward achieving meaningful progress toward reducing maternal mortality.

Although the Program did not initially plan to carry out demand-generation activities, these were incorporated in response to interim evaluations.

### 4. Develop a Deliberate, Structured Sustainability Strategy

While an intention to sustain the program was always part of the approach, the fourth and final lesson learned is the importance of developing a deliberate, structured sustainability strategy. The following domains played a key role in ensuring the Program's sustainability: (1) political support; (2) funding stability; (3) partnerships; (4) organizational capacity; (5) program evaluation and adaptation; and (6) communications. Specifically, the Program's foundation for sustainability included: implementation by Tanzanian teams, as opposed to teams from the Global North; embedding all interventions within existing government health system structures to foster strengthening from within; and working in partnership with all levels of government and in service of government plans and priorities. In the final 2 years of the Program, dedicated sustainability efforts were incorporated, including the creation of a regional mentorship team, budget advocacy, and the nurturing of both political and community-based champions committed to maintaining the Program once donor funding ceased. It was important to expand the partnership to include people and organizations who had political advocacy experience and knowledge of government budget processes. Only 2 budget cycles could be supported before the Program concluded. In hindsight, this activity should have started even sooner to mobilize sufficient domestic resources to sustain and scale the Program. When developing the sustainability strategy, Program partners looked to the global health literature for guidance^53^^–^^56^ but found little that was specific to maternal mortality reduction and FP programs.^57^ Our resulting approach to sustaining the Program, therefore, makes an important contribution to the field, one that will be augmented once a planned sustainability evaluation is conducted in 2022–2023. This follow-up evaluation will examine the facilitators of and barriers to the Program's long-term sustainability and is further evidence of the Program's commitment to inform public health practice in resource-poor settings.

The Program demonstrated that reducing maternal and perinatal mortality through the decentralization of lifesaving health services can work, and with sufficient resources and technical expertise, it could be a feasible approach in places with similar contexts. It is important to note that task sharing of obstetric surgery was already well accepted and supported in Tanzania at the start of the Program and may not be a strategy that can be quickly adopted in other countries. At the same time, the Program was constrained by external structural factors which were beyond the scope of this Program to address, such as shortages of human resources (especially the cadres most needed to deliver high-quality maternal and reproductive health services); pharmaceutical and medical supply stockouts; and social determinants that affect women's health throughout Kigoma. Collaboration with different government agencies and civil society organizations focused on addressing these structural factors might have allowed the Program to achieve more; it is a sobering reminder that health programming does not operate in a vacuum. Siloed approaches to development are limited in the impact they can have.

The Program was constrained by external structural factors; a sobering reminder that health programming does not operate in a vacuum.

## CONCLUSION

The multifaceted, 13-year Program in Kigoma made important progress toward improving the availability, access, and utilization of high-quality maternal and reproductive health services, resulting in significant reductions in maternal and perinatal mortality. By embedding the Program within the existing health system, and through the efforts made to build local capacity, the improvements are likely to be sustained. A follow-up evaluation in Kigoma in the coming years will explore the longer-term impact of the Program and, along with the implementation lessons described in this article, will provide critical information for others interested in replicating the Program and continuing the effort to save the lives of mothers and babies worldwide.

## Notes

**Peer Reviewed**
